# Modified Cytoplasmic Ca^2+^ Sequestration Contributes to Spinal Cord Injury-Induced Augmentation of Nerve-Evoked Contractions in the Rat Tail Artery

**DOI:** 10.1371/journal.pone.0111804

**Published:** 2014-10-28

**Authors:** Hussain Al Dera, Brid P. Callaghan, James A. Brock

**Affiliations:** 1 Department of Anatomy and Neuroscience, University of Melbourne, Victoria, Australia; 2 Basic Medical Sciences, College of Medicine, King Saud bin Abdulaziz University for Health Sciences, Riyadh, Saudi Arabia; University of Canberra, Australia

## Abstract

In rat tail artery (RTA), spinal cord injury (SCI) increases nerve-evoked contractions and the contribution of L-type Ca^2+^ channels to these responses. In RTAs from unoperated rats, these channels play a minor role in contractions and Bay K8644 (L-type channel agonist) mimics the effects of SCI. Here we investigated the mechanisms underlying the facilitatory actions of SCI and Bay K8644 on nerve-evoked contractions of RTAs and the hypothesis that Ca^2+^ entering via L-type Ca^2+^ channels is rapidly sequestered by the sarcoplasmic reticulum (SR) limiting its role in contraction. *In situ* electrochemical detection of noradrenaline was used to assess if Bay K8644 increased noradrenaline release. Perforated patch recordings were used to assess if SCI changed the Ca^2+^ current recorded in RTA myocytes. Wire myography was used to assess if SCI modified the effects of Bay K8644 and of interrupting SR Ca^2+^ uptake on nerve-evoked contractions. Bay K8644 did not change noradrenaline-induced oxidation currents. Neither the size nor gating of Ca^2+^ currents differed between myocytes from sham-operated (control) and SCI rats. Bay K8644 increased nerve-evoked contractions in RTAs from both control and SCI rats, but the magnitude of this effect was reduced by SCI. By contrast, depleting SR Ca^2+^ stores with ryanodine or cyclopiazonic acid selectively increased nerve-evoked contractions in control RTAs. Cyclopiazonic acid also selectively increased the blockade of these responses by nifedipine (L-type channel blocker) in control RTAs, whereas ryanodine increased the blockade produced by nifedipine in both groups of RTAs. These findings suggest that Ca^2+^ entering via L-type channels is normally rapidly sequestered limiting its access to the contractile mechanism. Furthermore, the findings suggest SCI reduces the role of this mechanism.

## Introduction

In spinal cord injured (SCI) patients with high thoracic or cervical lesions, spinal reflex-evoked vasoconstriction elicited by distension of visceral organs, or by some other unheeded afferent input from caudal to the lesion, is exaggerated leading to large increases in blood pressure (autonomic dysreflexia (AD); [1]). AD is normally attributed to excessive sympathetic nerve activity resulting from plasticity of connections within the damaged spinal cord [2]. However, in SCI subjects with AD, bladder compression or electrical stimulation of abdominal skin elicits only brief bursts of cutaneous vasoconstrictor nerve activity [3]. These bursts produced prolonged reductions in skin blood flow compared to those in able-bodied subjects [3], suggesting that the vasculature becomes hyperresponsive to sympathetic activity. Accordingly, in SCI rats that also display AD, neurovascular transmission is markedly increased in arteries isolated from both cutaneous and splanchnic vascular beds [4], [5], [6].

We have demonstrated that SCI markedly increases nerve-evoked constriction of rat tail artery and that this change is associated with an increase in sensitivity of these responses to inhibition by the L-type Ca^2+^ channel blocker nifedipine [6], [7]. Furthermore, in tail arteries from uninjured rats, we demonstrated that Ca^2+^ entry via L-type Ca^2+^ channels normally plays a relatively minor role in nerve-evoked contractions and that the enhancement of nerve-evoked contractions produced by the L-type Ca^2+^ channel agonist Bay K8644 mimics that produced by SCI [7]. Together these findings suggest that SCI augments nerve-evoked contractions by increasing the contribution of Ca^2+^ influx via L-type Ca^2+^ channels to contraction, but the mechanisms underlying this enhancement have not been determined.

Investigations of noradrenaline (NA) release from sympathetic nerve terminals in a range of tissues, including the rat tail artery, indicate that it is dependent primarily on Ca^2+^ entering through N-type Ca^2+^ channels and does not normally involve Ca^2+^ influx through L-type Ca^2+^ channels [8]. However, electrophysiological studies have demonstrated that postganglionic sympathetic neurons express functional L-type Ca^2+^ channels [9], [10] and stimulation of these channels with Bay K8644 can increase action potential-evoked NA release in rabbit ear artery [11]. Therefore an increase in neurotransmitter release could contribute to the augmentation of nerve-evoked contractions produced by Bay K8644.

The question remains why L-type Ca^2+^ channels, whose activity is increased by Bay K8644, normally play a relatively minor role in nerve-evoked contractions of the tail artery. Previous studies in tail artery have suggested that Ca^2+^ entering through L-type Ca^2+^ channels is rapidly sequestered by the sarcoplasmic reticulum (SR), limiting its access to the contractile machinery [12]. Therefore Bay K8644 by augmenting Ca^2+^ influx may overcome this Ca^2+^ buffering role of the SR, increasing access of Ca^2+^ entering via L-type Ca^2+^ channels to the contractile mechanism.

In the present study we investigated the mechanisms by which SCI and Bay K8644 increase nerve-evoked contractions and the possibility that reducing Ca^2+^ sequestration by the SR increases the contribution of L-type Ca^2+^ channels to these responses. The findings demonstrate that SCI does not change the size or gating of Ca^2+^ channel currents recorded in tail artery myocytes. In addition, the findings demonstrate that depleting the SR Ca^2+^ store with ryanodine or interrupting Ca^2+^ uptake into the SR with the SR Ca^2+^-ATPase (SERCA) inhibitor cyclopiazoinc acid selectively increases nerve-evoked contractions in tail arteries from sham-operated rats and also increases the sensitivity of these responses to nifedipine.

## Methods

All experimental procedures were approved by the University of Melbourne Animal Experimentation Ethics Committee and they conformed to the Australian Code of Practice for the Care and Use of Animals for Scientific Purposes.

### Spinal cord transection

The spinal cord was transected in 17 female Sprague Dawley rats (∼8 weeks of age; supplied by Biomedical Sciences Animal Facility, University of Melbourne) that were anaesthetized with isoflurane (2–3% in oxygen). A full description of the surgery is described in Al Dera et al [7] and is briefly outlined here. A laminectomy was performed to remove the dorsal aspect of the T10 vertebrum and the spinal cord transected at the T11 spinal level. This lesion severs all bulbospinal connections to the preganglionic neurons that control the tail artery, which are located in the T13-L2 spinal segments [13], [14]. The laminectomy site was closed and postoperatively animals received sterile saline (2 mL) and analgesic (0.06 mg kg^−1^ buprenorphine; Reckitt Benckiser, Sydney, Australia) by subcutaneous injection. Additional injections of saline and analgesic were administered daily for the first 3 post-operative days. Bladders were manually expressed 3 times daily until the animals regained the ability to empty their own bladders. In 17 age-matched sham-operated female rats, the laminectomy was performed to expose the spinal cord and post-operative treatments were similar except for bladder management. SCI and sham-operated rats were maintained for six weeks post surgery.

### Tissue collection

Rats were deeply anaesthetized with the volatile anesthetic isoflurane and killed by decapitation. The tail artery was dissected from 20–40 mm distal to the base of the tail. After isolation, arteries were maintained in physiological saline with the following composition (in mmol/L): Na^+^, 150.6; K^+^, 4.7; Ca^2+^, 2; Mg^2+^, 1.2; Cl^−^, 144.1; H_2_PO_4_^−^, 1.3; HCO_3_^−^, 16.3; glucose, 7.8. This solution was gassed with 95% O_2_ - 5% CO_2_ and warmed to 36–37°C.

### Mechanical responses

Segments of artery (∼1.5 mm long) were mounted isometrically between stainless steel wires (50 µm diameter) in two 4-chamber myographs (Multi Myograph 610 M, Danish Myo Technology, Denmark) as previously described [7]. As there was some variation in the lengths of the artery segments studied, contractions were measured as increases in wall tension (force/2×vessel length; [see 15]). After equilibration for 30–45 mins, contractions to phenylephrine (3 µM) were used to test the vascular smooth muscle function of arteries from sham-operated and SCI rats. We have previously demonstrated that SCI does not change the EC_50_ or maximum contraction of tail arteries to phenylephrine [6], [7]. In addition, carbachol (1 µM) induced vasorelaxation of phenylephrine constricted vessels was used to assess the vasorelaxatory action of activating the endothelium.

### Electrically evoked contractions

The perivascular nerves were electrically stimulated with trains of 100 pulses (20 V, 0.2 ms pulse width) at 1 Hz applied through platinum plate electrodes mounted either side of the tissue. In preliminary experiments, it was confirmed that these stimulus parameters were supramaximal for activating the perivascular sympathetic axons and that contractions produced by electrical stimulation were fully blocked by tetrodotoxin (0.5 µM), confirming they are mediated by action potential-evoked neurotransmitter release. In all experiments assessing the effects of drugs on nerve-evoked contractions, the effects of each added drug was assessed 25 mins after its addition.

### Electrochemical recording

The release of endogenous NA was monitored using continuous amperometry in tail arteries from unoperated rats as described previously [8], [16]. Briefly, artery segments ∼10 mm in length were pinned to the Sylgard (Dow-Corning) covered base of a 2 ml recording chamber that was perfused continuously at 3–5 ml min^−1^ with physiological saline. The proximal end of the artery was drawn into a suction stimulating electrode and the perivascular nerves were electrically activated (20 V, 0.2 ms pulse width). Recordings were made at a site 1–2 mm distal to the mouth of the suction electrode with a carbon fibre electrode (7 µm diameter) that was mounted so that the first 100–200 µm from the tip of the fibre was in contact with the adventitial surface of the artery. The electrode was connected to an AMU130 Nanoamperometer (Radiometer-Analytical SA, Villeurbanne Cedex, France) and a potential difference of +0.3 V was applied between the recording electrode and an Ag/AgCl pellet placed in the recording chamber medium. The current required to maintain this voltage was monitored.

In these experiments, the physiological saline contained the *α*_1_-adrenoceptor antagonist, prazosin (0.1 µM), to inhibit contractions due to released NA and the neuronal NA transporter inhibitor, desmethylimipramine (0.1 µM), to inhibit neuronal reuptake of released NA. During the experiments, the arteries were stimulated at 1 min intervals with 10 pulses at 10 Hz and Bay K8644 (0.1 µM) or cyclopiazonic acid (CPZ; 1 µM) was added to the superfusing solution following the 10^th^ train of stimuli and left in contact with the artery for a further 20 trains. At the end of the experiments, the Ca^2+^ channel blocker, Cd^2+^ (0.1 mM), was added to verify that the signals recorded were due to Ca^2+^-dependent release of NA [see 8]. As a positive control, S_2_/S_1_ ratios were also determined in tissues treated with the α_2_-adrenoceptor antagonist, idazoxan (0.1 µM), which increases NA release in the rat tail artery by interrupting α_2_-adenoceptor-mediated autoinhibition [see 16].

### Isolation of rat tail artery myocytes

After dissection, tail arteries were cut open lengthwise and divided into small segments (∼2 mm in length) in Hanks buffer (Ca^2+^ free; Sigma-Aldrich, Castle Hill, NSW, Australia). The segments were incubated in dispersion solution (Hanks buffer (Ca^2+^ free) containing 0.7 mg ml^−1^ collagenase Type IA (Sigma-Aldrich), 0.5 mg ml^−1^ protease Type XIV (Sigma-Aldrich) and 2 mg ml^−1^ bovine serum albumin (Sigma-Aldrich)) for 10–14 min at 37°C in a 5% CO_2_ incubator. The tissue was then rinsed 4 times using Hanks buffer containing 2 mg ml^−1^ BSA. Smooth muscle cells (SMCs) were dispersed by gentle trituration of the segments with a wide-tipped fire-polished Pasteur pipette. The cell suspension was stored in Hanks buffer containing bovine serum albumin (2 mg ml^−1^) and Ca^2+^ (0.1 mM) at 4°C and used within 8 h.

### Electrophysiology

Ba^2+^ currents (I_Ba_) in myocytes were measured using the perforated patch configuration. The bath solution used to record I_Ba_ was composed of (in mmol/L) NaCl 130, KCl 5.4, BaCl_2_ 10, glucose 10, EGTA 0.1 and HEPES 10 (pH adjusted to 7.4 with NaOH). The pipette solution for perforated patch recording contained (in mmol/L) CsMeSO_4_ 120, tetraethylammonium Cl 20, EGTA 1, and HEPES 20 (pH adjusted to 7.2 with CsOH). Nystatin (10 mg/mL) was dissolved in dimethylsulfoxide (DMSO), sonicated, and diluted to give a final concentration of 150 µg/mL in the pipette solution. Inward currents were measured at room temperature using an Axopatch-1D patch-clamp amplifier, digitized with a 16-bit analog to digital converter (Model DIGIDATA 1322, Axon Instruments), and controlled by pClamp8 (Axon Instruments).

### Data analysis

The output from the myograph and amperometer was recorded and analyzed using a PowerLab data acquisition system and the program Chart (ADInstruments, Bella Vista, NSW, Australia). Because the contractions to 100 pulses at 1 Hz have both an initial phasic and a later tonic component, the amplitude of these responses was measured at both the 10^th^ and 100^th^ pulse. As electrical stimulation produced an artefact (revealed in Cd^2+^) that lasted up to 500 ms following the last stimulus in the train, the amplitude of the NA-induced oxidation currents were measured 1 s following the last stimulus. The mean amplitudes of the three responses immediately before (S_1_) and 17–20 mins following the addition of Bay K8644 or CPZ (S_2_) were determined. The S_2_/S_1_ ratios in Bay K8644 or CPZ treated tissues were compared with those determined at the same points in control experiments with no drug added.

To construct I–V relationships, cells were voltage-clamped at −80 mV and then stepped from −80 mV to +60 mV in 10 mV increments evoked 20 seconds apart. Activation relationships for the Ca^2+^ channels were determined by calculating the peak conductance at each test potential by using the equation I_Ba_ = *g*_Ba_×(V–E_rev_), where *g*_Ba_, V, and E_rev_ are peak conductance, test potential, and reversal potential, respectively. A double-pulse protocol was used to measure inactivation of Ca^2+^ channel current as a function of membrane potential. Conditioning steps from −80 to 30 mV (in 10 mV intervals) were applied for 3.5 s. After a 25 ms step to −80 mV, the membrane potential was stepped to 0 mV for 250 ms. Resulting currents were normalized to the maximum current obtained after a conditioning potential of −80 mV (I/I_max_) and plotted as a function of the conditioning potential. The data were fitted by the Boltzmann equation using GraphPad Prism (GraphPad Software, Inc. CA, USA).

All statistical comparisons were made using SPSS 22 (IBM corporation, NY, USA. For experiments investigating the effects of drugs in control or SCI arteries comparisons were made with Student’s paired *t*-tests. When multiple effects were assessed in a single experiment, the data was first subjected to repeated measures ANOVA and the *P* values for pairwise comparisons were adjusted using the false discovery rate procedure [17]. Comparison between the measures in control and SCI arteries were made with either Student’s unpaired tests or Mann Whitney U-tests if the variation differed significantly between groups of data (assessed by Levine’s test). Data are presented as mean and standard error (SE) or as median and interquartile range (IQR) when the Mann Whitney U-test was used. *P* values <0.05 were considered to indicate significant differences. Unless otherwise indicated in the text, *P* values were obtained using unpaired *t*-tests. In all cases, *n* indicates the number of animals studied.

### Drugs

Bay K8644, nifedipine, phenylephrine HCl, prazosin, desmethylimipramine, carbachol, cyclopiazonic acid and nystatin were supplied by Sigma Aldrich (Castle Hill, NSW, Australia). Ryanodine was supplied by Tocris Bioscience (Bristol, UK). Drugs were prepared as stock solutions in water (phenylephrine, desmethylimipramine, carbachol), ethanol (nifedipine, Bay K8644) or DMSO (ryanodine, cyclopiazonic acid, prazosin). In all experiments, the highest concentration of ethanol or DMSO applied was 0.1% (w v^−1^).

## Results

### Basal conditions for the experiments investigating nerve-evoked contractions

Mechanical responses were recorded in artery segments from twelve sham-operated rats (control arteries) and twelve SCI rats (SCI arteries). The estimated lumen diameter of the artery segments after the normalization procedure (calculated from the measured lumen circumference) was significantly smaller for the SCI arteries (control: 831±29 µm; SCI: 737±20 µm; *P*<0.01). As a consequence the basal wall tension was larger for control arteries (4.3±0.2 mN mm^−1^) than it was for SCI arteries (3.7±0.2 mN mm^−1^; *P*<0.01). These effects of SCI are similar to those previously reported [6], [7]. The increase in wall tension produced by 3 µM phenylephrine did not differ between control and SCI arteries (control: 8.4±0.6 mN mm^−1^; SCI: 7.7±0.7 mN mm^−1^; *P* = 0.29). Furthermore, when contracted with phenylephrine (3 µM), the % relaxation produced when the endothelium was stimulated with carbachol (1 µM) did not differ between control (47±7%) and SCI arteries (58±5%; *P* = 0.1).

### Bay K8644 produces a smaller facilitation of nerve-evoked contractions in SCI arteries

**Figure 1:**
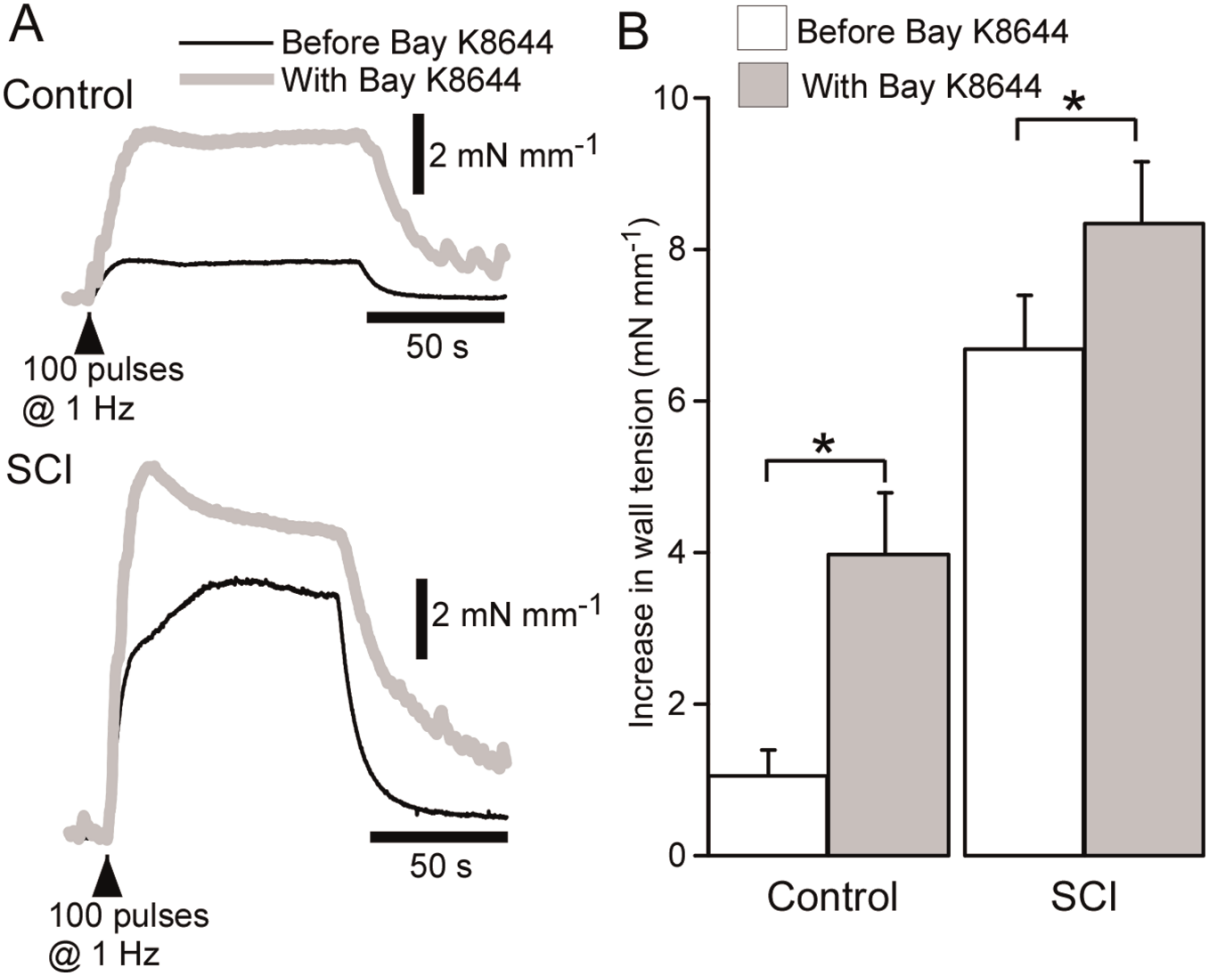
The facilitation of nerve-evoked contraction produced by Bay K8644 (0.1 µM) was greater in arteries from sham-operated rats (control arteries) than in arteries from spinal cord injured rats (SCI arteries). (**A**) Averaged traces showing contractions to 100 pulses at 1 Hz in control (*upper traces*) and SCI arteries (*lower traces*) before (*black line*) and during (*grey line*) application of Bay K8644 (0.1 µM). (**B**) Increases in wall tension measured at the 100^th^ pulse during the trains stimuli in control (*n* = 6) and SCI (*n* = 6) arteries before (*white bars*) and during (*grey bars*) application of Bay K8644. Data are presented as means and SEs and statistical comparisons were made with paired *t*-tests. *P<0.05.

Using tail arteries from unoperated rat we have previously reported that the L-type Ca^2+^ channel agonist Bay K8644 mimics the effects of SCI on nerve-evoked contractions. Here we investigated the facilitatory effects of Bay K8644 (0.1 µM) on contractions evoked by 100 pulses at 1 Hz in control (*n* = 6) and SCI (*n* = 6) arteries. As previously reported [6], [7], contractions of SCI arteries evoked by 100 pulses at 1 Hz were markedly larger than those of control arteries (**Fig. 1A, B** **;** *P*<0.01). In both groups of vessels, Bay K8644 increased the amplitude of contractions to this stimulus (**Fig. 1A, B**). However, the percentage increase in contraction amplitude measured at the 100^th^ pulse was much larger in control arteries (median 403%, IQR 194–609%) than SCI arteries (median 124%, IQR 117–138%, Mann Whitney U-test *P*<0.05). We have previously reported that the facilitatory effect of Bay K8644 on nerve-evoked contractions is completely blocked by nifedipine [7], confirming that it is mediated by an increase L-type Ca^2+^ channel activity. The finding that the facilitatory effect of Bay K8644 was much greater in control than SCI arteries may indicate that Ca^2+^ channel activity is elevated prior to adding this agent in SCI arteries.

### Bay K8466 does not increase NA release

**Figure 2:**
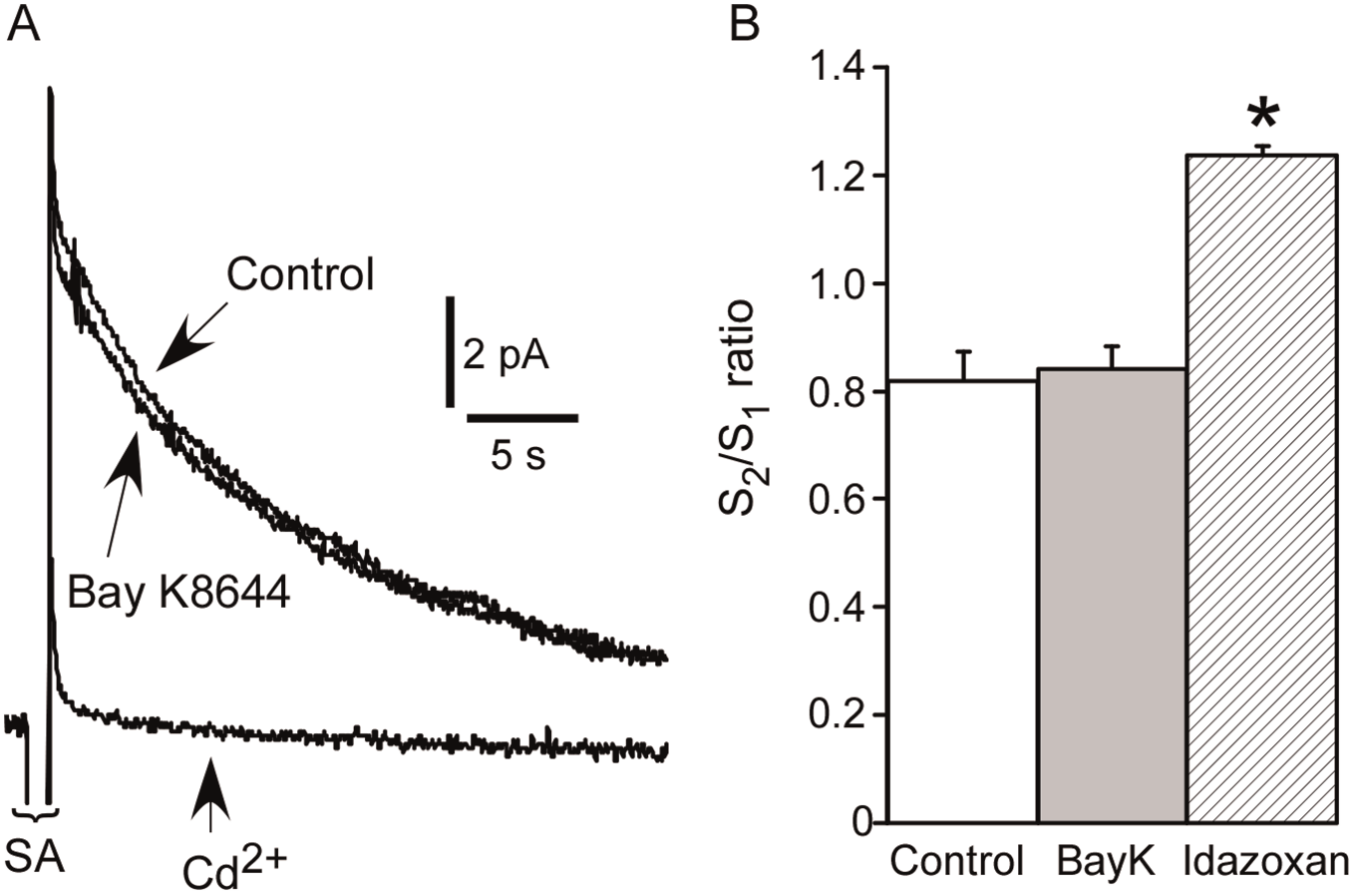
Bay K8644 did not change noradrenaline (NA)-induced oxidation currents evoked by nerve stimulation. (**A**) Overlaid averaged traces showing NA-induced oxidation currents that followed the stimulus artifacts (SA) before and during application of Bay K8644 (0.1 µM). In addition, (**A**) shows an averaged trace recorded during the subsequent addition of Cd^2+^ (0.1 mM) to block neurotransmitter release. (**B**) Mean stimulus period 2 to stimulus period 1 (S_2_/S_1_) ratios in tissues with Bay K8644 (BayK) added between the stimulus periods (*n* = 6; *grey bar*) and time-matched control tissues (*n* = 6; *white bar*). This graph also shows that S_2_/S_1_ ratios increased significantly in tissues treated with the α_2_-adrenoceptor antagonist idazoxan (Idaz) between the stimulus periods (0.1 µM, *n* = 6; *hatched bar*). The data are presented as means and SE and statistical comparisons with control were made with unpaired *t-*tests. *P<0.05.

*In situ* amperometry was used to assess the possibility that Bay K8644 (0.1 µM) augments nerve-evoked contraction by increasing NA release from the sympathetic nerve terminals. These experiments used tail artery segments from unoperated rats that were stimulated with trains of 10 pulses at 10 Hz. The stimulation artifacts during the periods of stimulation prevented NA-induced oxidation currents evoked by individual stimuli being discerned, but summation of these signals produced a slowly decaying oxidation current following the last stimulus in the train (**Fig. 2A**). In comparison with time-matched controls (*n* = 6), Bay K8644 (*n* = 6) did not change NA release (assessed by S_2_/S_1_ ratios, see methods) whereas blockade of prejunctional α_2_-adrenoceptors with idazoxan (0.1 µM, *n* = 6) significantly increased the NA release (**Fig. 2B**). The stimulus-evoked oxidation currents were abolished by the Ca^2+^ channel blocker Cd^2+^ (0.1 mM, **Fig. 2A**). These finding demonstrate that Bay K8644 does not increase NA release.

### The depolarization-evoked inward Ba^2+^ current did not differ between SMCs from SCI and control rats

**Figure 3:**
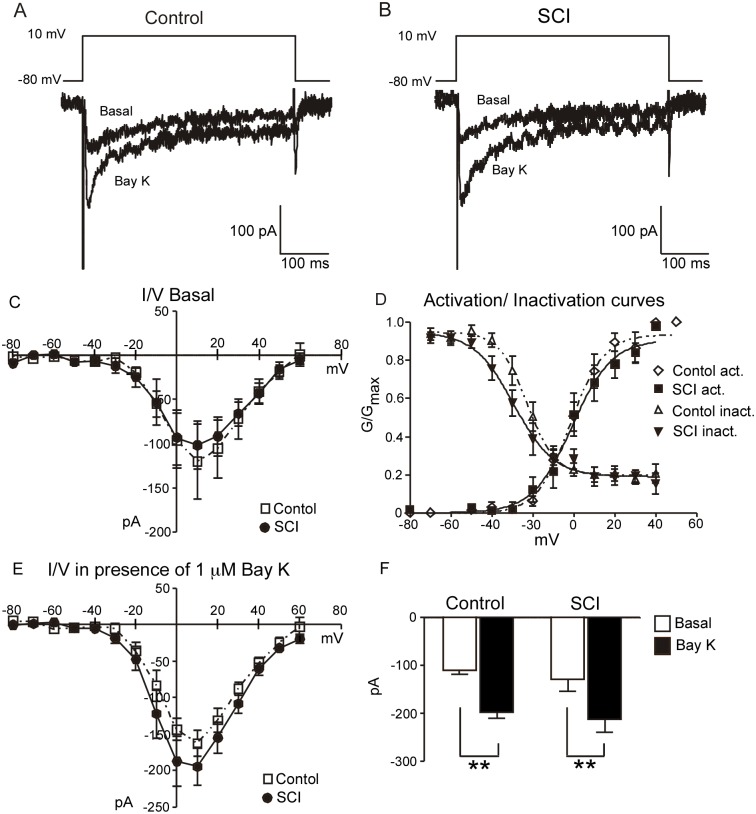
Calcium channel currents recorded from tail artery vascular smooth muscle cells (SMCs) are not changed by spinal cord injury (SCI). (**A, B**) Ba^2+^ currents elicited by a 250 ms depolarizing step to +10 mV from a holding potential of −80 mV in a representative SMC from a sham-operated (control) rat (**A**) and a SCI rat (**B**) before (basal) and during application of Bay K8644 (1 µM). (**C**) Leak-subtracted current-voltage relations in control and SCI SMCs under basal conditions (control: 8 cells, *n* = 5; SCI: 9 cells, *n* = 5). (**D**) Normalized conductance-voltage relations for currents from control and SCI SMCs. The activation and inactivation curves in each case are the least squares-fit to the Boltzmann equation. (**E**) Current-voltage relations in control and SCI SMCs in the presence of Bay K8644 (1 µM) (control: 8 cells, n = 5; SCI: 9 cells, n = 5). (**F**) Mean increase in current amplitude produced stepping from −80 mV to +10 mV in the absence and in the presence of Bay K8644 (control: 10 cells, *n* = 5; SCI: 9 cells, *n* = 5). The data are presented as means and SE and statistical comparisons were made with paired *t-*tests. **P<0.01.

Perforated patch recordings were used to determine if the depolarization-evoked Ba^2+^ currents carried by Ca^2+^ channels differed between SMCs isolated from control and SCI arteries. When tail artery SMCs are depolarized from −80 mV, it has been reported that ∼60% of the Ca^2+^ channel current carried by Ba^2+^ is sensitive to nifedipine (i.e. is due to activation of L-type Ca^2+^ channels; [18]). Inward currents had similar amplitudes in SMCs isolated from both groups of arteries (**Fig. 3A–C**) and the peak Ba^2+^ currents elicited at +10 mV did not differ significantly (control: −3.07±0.2 pA/pF, 8 cells, *n* = 5; SCI: −3.44±0.5 pA/pF, 9 cells, *n* = 5; *P* = 0.62). Membrane capacitance also was similar (control: 38.94±2.3 pF; SCI: 36.6±1.72 pF; *P* = 0.60). Voltage pulse protocols were used to obtain the activation and inactivation curves for control and SCI SMCs (**Fig. 3D**). These analyses provided similar values for the half-maximal (V_1/2_) current activation (control: −1.7±1.6 mV; SCI: −0.8±2.3 mV; *P* = 0.70) and inactivation (control: −22.6±1.5 mV; SCI: −29.27±2.4 mV; *P* = 0.73) in control and SCI SMCs. In addition, in both groups of SMCs, application of Bay K8644 (1 µM) approximately doubled the amplitude of Ba^2+^ currents measured at 10 mV (**Fig. 3E, F**), consistent with their primarily being mediated by L-type Ca^2+^ channels. These findings indicate that SCI does not detectably change Ca^2+^ channel density in SMCs or modify the gating of these channels.

### Interrupting Ca^2+^ sequestration by the sarcoplasmic reticulum selectively increased the size of nerve-evoked contractions in control arteries

**Figure 4:**
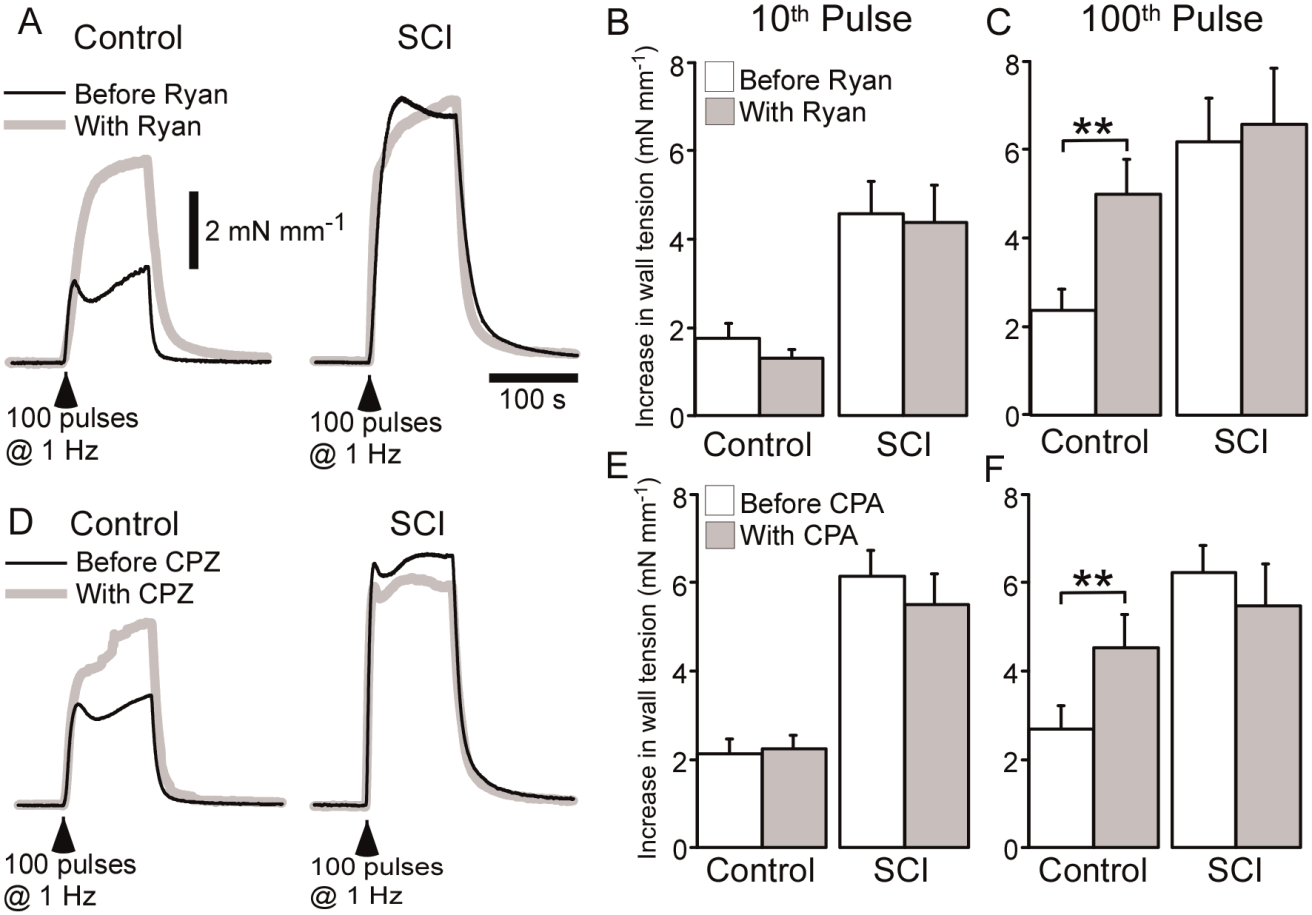
Both ryanodine (Ryan; 10 µM) and cyclopiazonic acid (CPZ; 1 µM) increased the amplitude of nerve-evoked contractions in arteries from sham-operated rats (control arteries) but not in those from spinal cord injured rats (SCI arteries). (**A, D**) Averaged traces showing contractions to 100 pulses at 1 Hz in control (*left traces*) and SCI arteries (*right traces*) before (*black line*) and during (*grey line*) application of ryanodine (**A**) or CPZ (**D**). (**B, C, E, F**) Increases in wall tension measured at the 10^th^ (**B, E**) and 100^th^ pulse (**C, F**) during the trains of stimuli in control (*n* = 6) and SCI (*n* = 6) arteries before (*white bars*) and during (*grey bars*) application of ryanodine (**B, C**) or CPZ (**E, F**). Data are presented as means and SEs and statistical comparisons were made with paired *t*-tests. **P<0.01.

The possibility that a reduction in Ca^2+^ sequestration by the SR contributes to the augmentation nerve-evoked contractions in SCI arteries was investigated by assessing the effects of depleting the ryanodine-sensitive Ca^2+^ store (with 10 µM ryanodine) and by inhibiting Ca^2+^ uptake into the SR with the SERCA inhibitor CPZ (1 µM). In these experiments, the tissues were stimulated with trains of 100 pulses at 1 Hz. In control arteries, both ryanodine (*n* = 6) and CPZ (*n* = 6) did not change the size of contractions measured at the 10^th^ pulse during the trains of stimuli (**Fig. 4B, E**), but increased those measured at the 100^th^ pulse (**Fig. 4A, C, D, F**). This effect of ryanodine and CPZ was not observed in SCI arteries (*n* = 6), where neither agent changed the size of nerve-evoked contractions (**Fig. 4** **A, C, D, F**). Importantly, in the presence of either ryanodine or CPZ, the size of the contractions measured at the 100^th^ pulse did not differ significantly between control and SCI arteries (**Fig. 4C, F**; Ryanodine *P* = 0.31, CPZ *P* = 0.45). Because both ryanodine and CPZ selectively increased the size of nerve-evoked contractions in control arteries, these findings suggest that reduced Ca^2+^ sequestration by the SR contributes to the augmentation nerve-evoked contractions in SCI arteries.

A possible alternative explanation for the increase in nerve-evoked contraction produced by ryanodine and CPZ is if they act at a prejunctional site to increase neurotransmitter release. Previously it has been reported that 10 µM ryanodine does not change NA release in the rat tail artery [19]. However, 5 µM CPZ has been reported to produce a small (∼20%) increase in NA release in this artery [20]. To test whether 1 µM CPZ increases NA release in arteries from unoperated rats we used *in situ* amperometry. In comparison with S_2_/S_1_ ratios for NA-induced oxidation currents in time-dependent controls (0.84±0.04, *n* = 6), those in tissues that were treated with CPZ did not differ significantly (0.90±0.03, *n* = 6; *P* = 0.27). Therefore a prejunctional site of action for ryanodine and CPZ can be excluded in the present study.

### Both ryanodine and CPZ increased the blockade of nerve-evoked contractions produced by nifedipine

**Figure 5:**
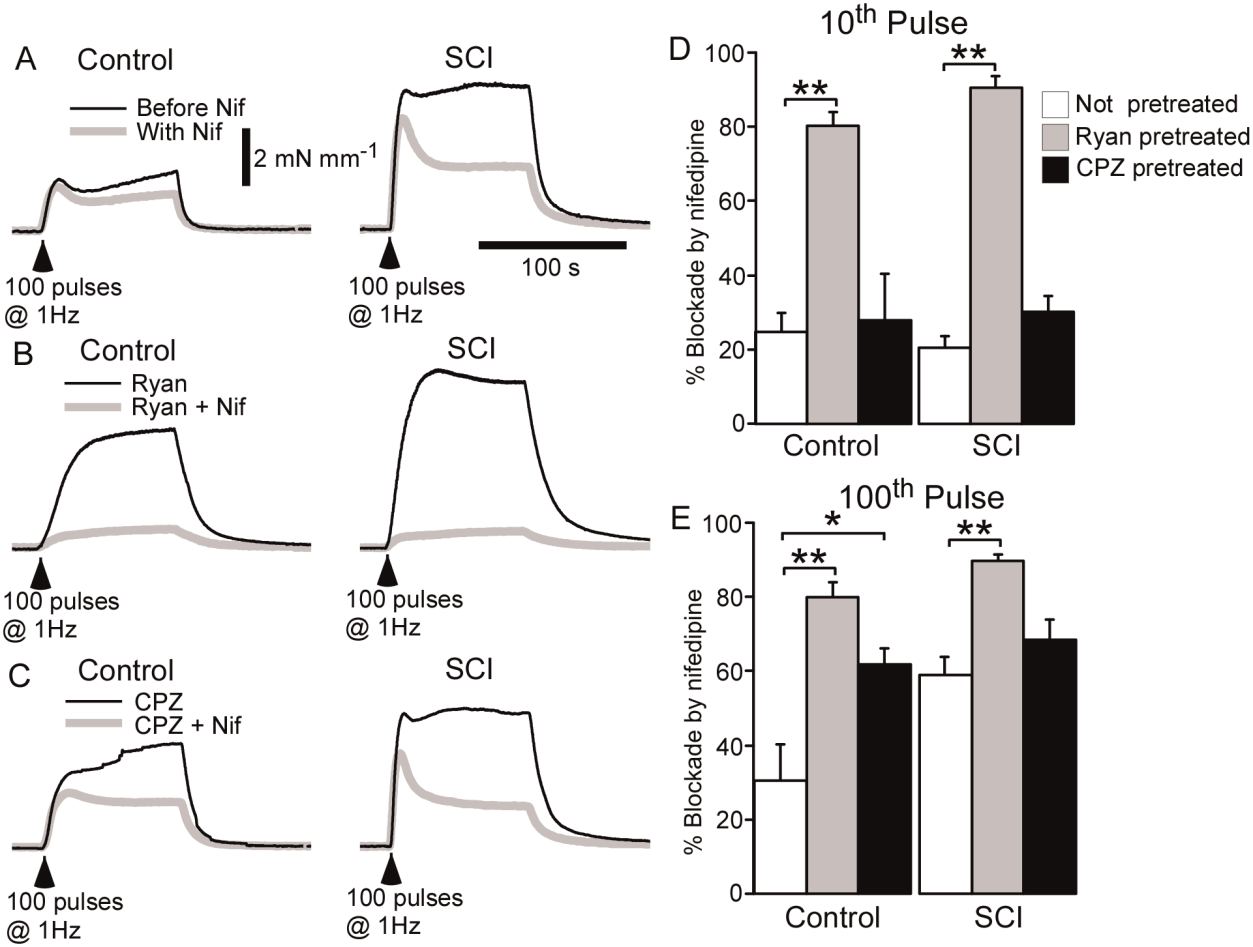
Both ryanodine (Ryan; 10 µM) and cyclopiazonic acid (CPZ; 1 µM) increased the blockade of nerve-evoked contractions produced by nifedipine (Nif; 1 µM) in arteries from sham-operated rats (control arteries). By contrast, only ryanodine increased the blockade of nerve-evoked contractions produced by nifedipine in arteries from spinal cord injured rats (SCI arteries). (**A–C**) Averaged overlaid traces showing contractions to 100 pulses at 1 Hz in control (*left traces*) and SCI arteries (*right traces*) in absence (**A**; *black line*) or in the presence of ryanodine (**B**; *black line*) or CPZ (**C**; *black line*) and following addition of nifedipine (*grey lines*). (**D, E**) The % blockade of contractions produced by nifedipine at the 10^th^ (**D**) and 100^th^ pulse (**E**) during the trains of stimuli in control (*n* = 6) and SCI (*n* = 6) arteries in the absence (*white bars*) or in the presence of ryanodine (*grey bars*) or CPZ (*black bars*). Data are presented as means and SEs and statistical comparisons were made with unpaired *t*-tests. *P<0.05, **P<0.01.

To test the hypothesis that Ca^2+^ sequestration by the SR limits the contribution of Ca^2+^ entering via L-type Ca^2+^ channels to nerve-evoked contraction, the effects of nifedipine (1 µM) were investigated in absence and in the presence of either ryanodine (10 µM) or CPZ (1 µM). In the absence of these agents, the % blockade of contractions produced by nifedipine at 10^th^ pulse during trains of stimuli at 1 Hz was similar in control and SCI arteries (**Fig. 5D**; *P* = 0.48). By contrast, at the 100^th^ pulse, the % blockade produced by nifedipine was greater in SCI arteries than in control arteries (**Fig. 5E**; *P*<0.05). The absolute size of the nifedipine-sensitive component of contraction at the 100^th^ pulse was also larger in SCI arteries (**Fig. 1A** **,** **Table 1**). The nifedipine-resistant contractions of SCI arteries measured at the 10^th^ pulse were still larger than control, whereas those measured at 100^th^ pulse had a tendency to be larger but this difference was not statistically significant (**Fig. 5A** **,** **Table 1**).

**Table 1:** The nifedipine-sensitive and nifedipine-resistant components of contractions evoked by 100 pulses at 1 Hz in control and SCI arteries with no pretreatment or pretreated with ryanodine (Ryan; 10 µM) or cyclopiazonic acid (CPZ; 1 µM).

		10^th^ Pulse		100^th^ Pulse	
		Nifedipine-sensitivecontraction	Nifedipine-resistantcontraction	Nifedipine-sensitivecontraction	Nifedipine-resistantcontraction
		mN mm^–1^	mN mm^–1^	mN mm^–1^	mN mm^–1^
Not pretreated	Control (n = 6)	0.44 ± 0.09	1.23 (1.02–1.68)	0.79 ± 0.35	1.34 (1.10–1.73)
	SCI (n = 6)	1.10 ± 0.18	4.59 (3.64–4.97)	3.14 ± 0.46	2.25 (1.59–2.74)
	*P* value	<0.01*	<0.01*	<0.01*	0.17
Ryan pretreated	Control (n = 6)	1.13 (0.73–1.39)	0.21 ± 0.04	3.65 ± 0.58	0.67 ± 0.11
	SCI (n = 6)	3.86 (2.54–5.90)	0.33 ± 0.09	5.56 ± 1.12	0.58 ± 0.15
	P value	<0.05*	0.24	0.16	0.63
CPZ pretreated	Control (n = 6)	0.56 ± 0.28	1.69 ± 0.36	2.27 ± 0.47	1.67 ± 0.30
	SCI (n = 6)	1.55 ± 0.27	3.65 ± 0.53	3.55 ± 0.67	1.53 ± 0.39
	*P* value	<0.05*	<0.05*	0.15	0.79

Data are presented as mean ± SE or median and interquartile range (in parentheses) and comparisons between control and SCI values were made with Student’s unpaired *t*-tests or Mann Whitney U-tests respectively (* indicates significant *P* values).

In ryanodine-pretreated control (*n* = 6) and SCI (*n* = 6) arteries, the % blockade of nerve-evoked contractions produced by nifedipine was increased at both the 10^th^ and 100^th^ pulse (**Fig. 5** **D, E**). At the 10^th^ pulse in the presence of ryanodine, the absolute size of the nifedipine-sensitive component of contraction was larger in SCI arteries than in control arteries (**Fig. 5B** **,** **Table 1**). By contrast, at the 100^th^ pulse, the absolute size of the nifedipine-sensitive component in the presence of ryanodine did not differ between control and SCI arteries (**Fig. 5B** **,** **Table 1**). The nifedipine-resistant component of contraction did not differ between the ryanodine-treated control and SCI arteries at either the 10^th^ and 100^th^ pulse (**Fig. 5B** **,** **Table 1**).

CPZ did not significantly change the % blockade of contractions produced by nifedipine at the 10^th^ pulse in both control (*n* = 6) and SCI arteries (*n* = 6; **Fig. 5D**). At the 100^th^ pulse, CPZ selectively increased the % blockade of contractions by nifedipine in control arteries (**Fig. 5E**). At this time point the absolute size of the nifedipine-sensitive and nifedipine-resistant components of contraction did not differ between control and SCI arteries (**Fig. 5C** **,** **Table 1**). Together these findings indicate that disruption of Ca^2+^ buffering by the SR increases the contribution of Ca^2+^ influx via L-type Ca^2+^ channels to nerve-evoked contractions.

## Discussion

The primary finding of this study is that the increased sensitivity of nerve-evoked contractions of tail arteries from SCI rats to nifedipine is not explained by an increase in functional membrane expression of L-type Ca^2+^ channels in SMCs. Furthermore, the greater augmentation of nerve-evoked contractions produced by Bay K8644 in control arteries does not appear to be explained by a greater stimulatory effect of this agent on L-type Ca^2+^ channel activity. Instead, the selective augmentation of nerve-evoked contractions produced by ryanodine and CPZ in control arteries, together with their greater sensitivity to nifedipine in the presence of these agents, suggests that Ca^2+^ entering via L-type Ca^2+^ channels is normally rapidly sequestered into the SR limiting its access to the contractile mechanism. As neither ryanodine nor CPZ increased nerve-evoked contractions of SCI arteries, we assume that Ca^2+^ entering via L-type Ca^2+^ channels is less susceptible to sequestration by the SR in these vessels and more freely accesses the contractile mechanism.

Bay K8644 did not change the amplitude of nerve-evoked NA-induced oxidation currents, which provide a real time measure of NA release [8]. This finding accords with previous reports that Bay K8644 does not change NA release from sympathetic nerve terminals in other rat tissues [21], [22]. As nerve-evoked contractions of both control and SCI arteries are mediated almost entirely by NA [6], [7], the facilitatory action of Bay K8644 on these responses cannot be attributed to increased neurotransmitter release from the sympathetic nerve terminals.

The size of the whole cell Ba^2+^ current carried by voltage-activated Ca^2+^ channels did not differ between SMCs from control and SCI tail arteries. There was also no difference in the voltage for half-maximal activation and inactivation of the Ba^2+^ current between the two groups of SMCs. When depolarized from −80 mV most of the Ba^2+^ current in tail artery SMCs is carried by L-type Ca^2+^ channels but there is also a component that is carried by T-type Ca^2+^ channels [18], [23]. Bay K8644 selectively enhances the L-type Ca^2+^ channel current in rat tail artery [23], [24]. In both control and SCI SMCs, the increase in Ba^2+^ current produced by BayK8644 was similar, consistent with there being a similar contribution of L-type Ca^2+^ channel activation to the increase in Ca^2+^ conductance. Together these findings indicate that increased L-type Ca^2+^ channel expression in SMCs is unlikely to explain the greater contribution of these ion channels to neural activation of SCI tail arteries. The findings also indicate that the greater augmentation of nerve-evoked contractions produced by Bay K8644 in control arteries, compared to SCI arteries, is not explained by greater stimulation of L-type Ca^2+^ channel activity in these vessels.

In control arteries, but not SCI arteries, both ryanodine and CPZ increased the size of nerve-evoked contractions measured at the end of the trains of 100 pulses at 1 Hz. As Bay K8644 increased the size of contractions in SCI arteries, the failure of ryanodine and CPA to increase these responses cannot be attributed to the muscle already being maximally activated. At the 100^th^ pulse, both ryanodine and CPZ also produced a greater increase the % blockade of nerve-evoked contractions by nifedipine in control arteries. As a consequence, while in the absence of these agents the absolute size of the nifedipine-sensitive component of contraction at the 100^th^ pulse was much larger in SCI arteries than in control arteries, in their presence it did not differ between these groups of vessels. These findings indicate that impairing the ability of the SR to accumulate and/or release Ca^2+^ increases the contribution of L-type Ca^2+^ channels to nerve-evoked contractions. Furthermore, as ryanodine and CPZ more markedly increased the contribution of L-type Ca^2+^ channels to neural activation of control arteries, this suggests that in these vessels Ca^2+^ entering via L-type Ca^2+^ channels is more susceptible to regulation by the SR than in SCI arteries.

It has previously been demonstrated that ryanodine does not change NA release in the tail artery [19] or other sympathetically innervated tissues in the rat [25], [26]. While it has been reported that CPZ increases NA-release in the tail artery [20], in the present study this effect was not observed. A likely explanation is that Tsai et al. [20] used 5 µM CPZ whereas 1 µM CPZ was used in the present study. Therefore the augmentation of nerve-evoked contractions produced by ryanodine and CPZ cannot be attributed to an increase in neurotransmitter release.

Ryanodine at 10 µM depletes Ca^2+^ from the SR by locking the ryanodine receptor channels open [27]. This action of ryanodine inhibits the contribution of Ca^2+^-induced Ca^2+^ release to activation of vascular muscle. In addition, the leak of Ca^2+^ from the SR induced by ryanodine prevents it from storing any Ca^2+^ accumulated by SERCA [27]. While both ryanodine and CPZ did not change the amplitude of contractions measured at the 10^th^ pulse, ryanodine selectively and similarly increased the % blockade of contraction produced by nifedipine in both control and SCI arteries. Furthermore in the presence of ryanodine, the small nifedipine-resistant contractions measured at the 10^th^ pulse did not differ between the groups of arteries. This selective action of ryanodine at the 10^th^ pulse suggests that Ca^2+^-induced Ca^2+^ release contributes to the early phase of contraction in the rail artery. A similar suggestion has been made in rat mesenteric arteries where the early phasic contraction to long trains of nerve stimuli was inhibited by ryanodine but this agent did not affect the later tonic phase of contraction [25].

Previously it has been reported that both ryanodine and CPZ increase the contribution of Ca^2+^ influx via L-type Ca^2+^ channels to activation of tail artery smooth muscle [12]. To explain this finding Nomura and Asano [12] suggested that Ca^2+^ entering the cell via L-type Ca^2+^ channels is rapidly sequestered into the SR limiting its access to the contractile machinery (see also [28]). As CPZ did not change nerve-evoked contractions of SCI arteries or their sensitivity to nifedipine, it is possible that the activity of SERCA in these vessels is lower than in control arteries. This change would explain the greater sensitivity of SCI arteries to nifedipine. An increase in the contribution of L-type Ca^2+^ channels to contraction following depletion of the intracellular Ca^2+^ stores has also been demonstrated in rat mesenteric veins [29]. In SMCs from rat mesenteric veins, intracellular Ca^2+^ chelation with BAPTA increased L-type Ca^2+^ channel activity recorded in cell attached patches. Therefore Thakali et al. [29] concluded that Ca^2+^ released from the intracellular stores normally inactivates L-type Ca^2+^ channels. This process of inactivation is mediated via the Ca^2+^ sensor protein calmodulin, which binds to the intracellular C terminus of the Cav1.2 α_1_-subunit to cause a conformational change that enables channel inactivation [30], [31]. Perhaps the increase in the nifedipine-sensitive contraction produced by ryanodine at the 10^th^ pulse in both control and SCI arteries indicates that Ca^2+^ released from a ryanodine-sensitive store normally inhibits L-type Ca^2+^ channel activity at this time point.

The effects of SCI on sympathetic nerve-mediated activation of the tail artery are similar to those produced when the postganglionic sympathetic neurons supplying this vessel are decentralized by lesioning their preganglionic inputs [32]. Therefore the changes in neurovascular function produced by SCI are almost certainly caused by the decrease in ongoing sympathetic nerve activity that is known to occur caudal to a spinal cord lesion [33]. The effects of decentralization on regulation of intracellular Ca^2+^ in vascular muscle cells are unknown. In rat vas deferens, the increase in smooth muscle reactivity produced by denervation is associated with a reduction in both SERCA Ca^2+^ uptake activity and protein expression of SERCA2 [34]. Down-regulation of SERCA has also been reported in denervated skeletal muscle [35]. In rat vas deferens homogenates, denervation also reduced the density of ryanodine binding sites but not their affinity [34]. As the effects of decentralization and denervation on smooth muscle reactivity are similar in the rat tail artery [36], it is possible that they are mediated, at least in part, by a down-regulation of SR proteins involved in intracellular Ca^2+^ homeostasis.

**Figure 6:**
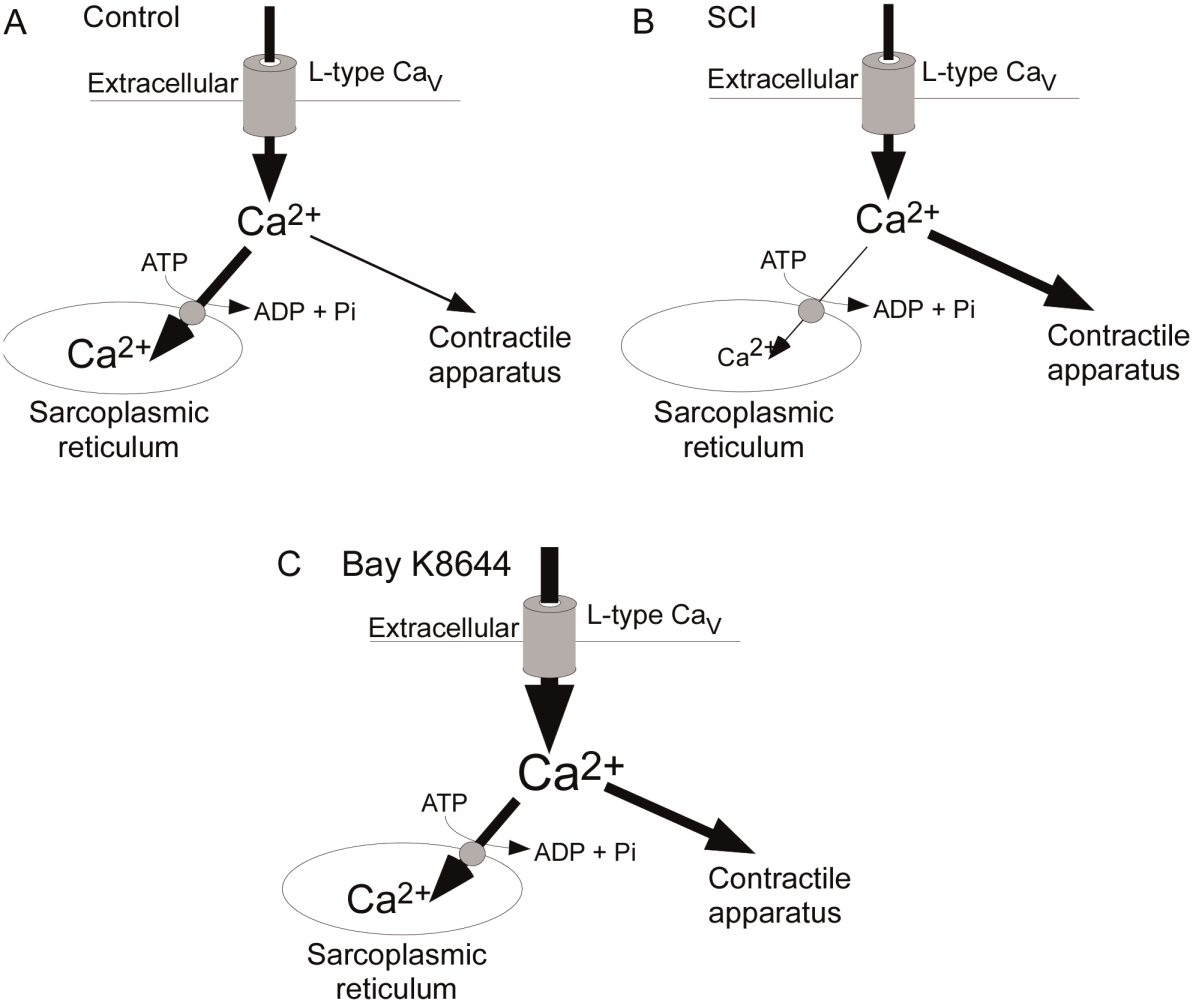
Schematic representations of the mechanisms regulating the contribution of Ca^2+^ influx via L-type Ca^2+^ channels to nerve-evoked contractions. (A) In control arteries, much of the Ca^2+^ entering the cell via L-type Ca^2+^ channels is rapidly sequestered into the sarcoplasmic reticulum (SR) limiting its access the contractile mechanism. (B) In SCI arteries, less of the Ca^2+^ entering through L-type Ca^2+^ channels is sequestered into the SR increasing its access to the contractile mechanism. (C) Bay K8644 increases Ca^2+^ entry via L-type Ca^2+^ channels overcoming the Ca^2+^ buffering capacity of the SR.

In conclusion, the increase in both nerve-evoked contraction amplitude and sensitivity to nifedipine produced by disrupting cytoplasmic Ca^2+^ buffering by the SR in control arteries suggests that Ca^2+^ entering via L-type Ca^2+^ channels is normally rapidly sequestered, reducing its access to the contractile mechanism (**Fig. 6A**). As nerve-evoked contractions of SCI arteries were larger and more sensitive to nifedipine, and disruption of Ca^2+^ buffering did not increase the size of these responses, it appears that sequestration of Ca^2+^ entering via L-type Ca^2+^ channels is lower in these vessels (**Fig. 6B**). If this interpretation is correct, the more marked facilitatory effect Bay K8644 on nerve-evoked contraction in control arteries is readily explained by the increase in Ca^2+^ influx overcoming the Ca^2+^ buffering role of the SR (**Fig. 6C**). In vascular muscle, there is also evidence that Ca^2+^ released from intracellular stores inhibits the activity of L-type Ca^2+^ channels and that this is prevented by impairing ability of the SR to accumulate and release Ca^2+^ [29]. Therefore this mechanism could provide an alternative explanation for the effects of ryanodine and CPZ observed in the present study.
